# A dosimetric comparison of non-coplanar volumetric modulated arc therapy and non-coplanar fixed field intensity modulated radiation therapy in hippocampus-avoidance whole-brain radiation therapy with a simultaneous integrated boost for brain metastases

**DOI:** 10.3389/fonc.2024.1428329

**Published:** 2025-01-23

**Authors:** Huaqu Zeng, MinZhi Zhong, Zongyou Chen, Shukui Tang, Zunbei Wen

**Affiliations:** ^1^ Radiotherapy Center, Gaozhou People’s Hospital, Gaozhou, China; ^2^ Department of Radiology, Guangzhou Red Cross Hospital, Guangzhou, China

**Keywords:** hippocampus sparing, brain metastases, simultaneous integrated boost, whole brain radiotherapy, volumetric modulated arc therapy, intensity modulated radiotherapy

## Abstract

**Objective:**

The aim of this study was to investigate the dosimetric differences between non-coplanar volumetric modulated arc therapy (VMAT) and non-coplanar fixed-field intensity-modulated radiotherapy (IMRT) in hippocampus-avoidance whole-brain radiation therapy with a simultaneous integrated boost (HA-WBRT+SIB) for brain metastases using the Monaco treatment planning system (TPS).

**Method:**

A total of 22 patients with brain metastases were retrospectively enrolled. Two radiotherapy treatment plans were designed for each patient: non-coplanar VMAT and non-coplanar fixed field IMRT. The dose distribution of targets and organs at risk (OAR), the number of monitor units (MUs), and pre-treatment plan verification were compared between the two plans while meeting the prescribed dose requirements of the target volume.

**Results:**

There were no significant differences in V_50_, V_55_, D_max_, heterogeneity index (HI) and conformity index (CI) of target PGTV between the two plans (*p*>0.05). For PTV-brain-SIB, there was no significant difference in D_98%_ between IMRT and VMAT (*p*=0.103). VMAT significantly improved the V_30_ of PTV-brain-SIB (*p*<0.001), decreased HI (*p*=0.003), and increased CI (*p*<0.001). There were no significant differences in the D_max_ to the brain stem, left and right lens, optic chiasm, pituitary gland, and left and right hippocampus between the two plans (*p*>0.05). Compared with IMRT, VMAT significantly reduced the D_max_ to the left and right eyes (*p*<0.001) and significantly increased the D_max_ to the right inner ear (*p*=0.010). There was no significant difference in the D_max_ to the left inner ear between VMAT and IMRT (*p*=0.458). Compared with IMRT, VMAT significantly reduced the D_max_ to the left optic nerve (*p*=0.006), but significantly increased the D_max_ to the right optic nerve (*p*=0.001). There was no significant difference in the D_max_ to the left and right hippocampus between VMAT and IMRT (*p*>0.05), but VMAT significantly increased the D_100%_ (*p*<0.05) compared with IMRT. Compared with VMAT, IMRT significantly reduced the MU (*p*<0.001) but VMAT has a higher treatment efficiency than IMRT, with an average reduction of 41 seconds (294.1 ± 16.4 s for VMAT, 335.8 ± 34.9 s for IMRT, *p*<0.001). Under the conditions of 3%/2 mm, and 2%/2 mm, the gamma passing rate of the IMRT QA was improved compared to VMAT, with an average increase of 0.6%, *p*=0.013, and 1.7%, *p*<0.001, respectively.

**Conclusion:**

Both non-coplanar VMAT and non-coplanar fixed field IMRT based on the Monaco TPS produce clinically acceptable results for HA-WBRT+SIB in patients with brain metastases. Compared with IMRT, VMAT has better dose distribution in the target volume and treatment efficiency, but IMRT can better protect the hippocampus and reduce the number of MUs.

## 1 Introduction

Over the past few years, the incidence rate of brain metastases has increased consistently (1). Whole brain radiotherapy (WBRT) for treating brain metastases, prophylactic cranial irradiation for treating small cell lung cancer, and cranial or craniospinal irradiation for treating malignant tumors of the central nervous system in children have all demonstrated clinical efficacy; however, they also increase cognitive neurotoxicity (2–6). Radiation-induced hippocampal damage plays a significant role in the decline of neurocognitive abilities in patients after WBRT (7). The hippocampus is a central element in memory formation, and the degree of atrophy and cognitive deficits are dependent on the delivered dose; thus, maximal protection of the hippocampus is imperative. Furthermore, the risk of brain metastases occurring in the hippocampus is below 5%; this also suggests that hippocampal avoidance during WBRT (HA-WBRT) is safe (8). Therefore, Radiation Therapy Oncology Group (RTOG) report 0933 proposed hippocampal protection during WBRT (9).

The combination of WBRT and a simultaneous integrated boost (SIB) of localized lesions for brain metastases has been shown to have advantages in terms of shortening treatment time, prolonged local control time, and overall survival (10). For patients with non-small cell lung cancer brain metastases, WBRT combined with a stereotactic radiotherapy boost or simultaneous boost may improve their survival rate compared to WBRT alone (11, 12). With the development of radiation therapy techniques, especially the advent of intensity-modulated radiation therapy (IMRT) techniques, it has become possible to protect organs-at-risk (OARs) such as the hippocampus during WBRT for brain metastases (13). IMRT has significant benefits in hippocampal protection for primary brain tumors, preventing neurocognitive decline and reducing the average dose to the hippocampus. Even after 6 months of follow-up post-irradiation, a neurocognitive benefit was seen in most patients (14). Many researchers have studied whether IMRT or volumetric modulated arc therapy (VMAT) alone in HA-WBRT protect the hippocampus during brain metastases and have confirmed that IMRT or VMAT can effectively protect the hippocampus in HA-WBRT (15–18).

Ilinca Popp et al. confirmed that hippocampus-avoidance whole-brain radiation therapy with a simultaneous integrated boost (HA-WBRT+SIB) could be an efficient therapeutic option for patients with multiple brain metastases. It is associated with improved local tumor control of existing metastases, higher intracranial progression-free survival, reduced death rates associated with neurological conditions, and an acceptable risk of radiation necrosis (19). HA-WBRT+SIB is a complex treatment regimen for patients with brain metastases, aimed at reducing adverse neurocognitive effects while increasing tumor control (19).

Developing an effective hippocampal protection plan for HA-WBRT+SIB treatment poses a challenge. Johannes Kraft et al. compared the dose delivered by the Varian Halcyon linear accelerator based on the Eclipse treatment planning system (TPS) and that delivered by the Elekta Synergy linear accelerator based on the Pinnacle TPS for HA-WBRT+SIB using VMAT. The whole-brain prescribed dose was 30 Gy, and the local boost was 51 Gy administered in 12 fractions. In their study, a 7 mm expansion around the hippocampus was implemented to form the hippocampal avoidance region (HAR).The Halcyon and Synergy Agility linear accelerators produced clinically comparable treatment plans for HA-WBRT+SIB in patients with multiple brain metastases (20). R. Vysakh et al. compared the dose distributions of the coplanar jaw-fixed VMAT (fVMAT) and the conventional coplanar VMAT without the jaw fixed based on the Versa HD linear accelerator and the Monaco TPS for HA-WBRT. With a whole-brain irradiation dose of 30 Gy in 10 fractions, and a 5 mm extracorporeal expansion of the hippocampus forming the HAR, they found that the Elekta Agility™ collimator system and the Monaco TPS can generate superior HA-WBRT plans using the fVMAT technique (21). Xie Xin et al. compared the dosimetric differences between the coplanar dynamic IMRT (dIMRT) and coplanar VMAT plans of the Varian Linear accelerator on the Eclipse TPS in HA-WBRT alone. They found that the hippocampal dose of the dIMRT group was superior to that of the VMAT group, but neither met the standard of RGOT 0933 (22). Fangyu Liu et al. evaluated the potential of the flattening filter-free (FFF) mode of a linear accelerator for patients with HA-WBRT in comparison with the flattened beams (FF) technique in the application of VMAT and IMRT using dosimetric and radiobiological indexes based on the volume of the hippocampus and target. Their study suggests that the FFF mode is feasible and advantageous in HA-WBRT and VMAT-FFF is the optimal solution in terms of dose distribution of the target, sparing OARs, probability of normal tissue complications of the hippocampus,and delivery efficiency compared to the other three techniques. Additionally, the advantages of the FFF technique for VMAT are more prominent in cases with small hippocampal volumes (33).

No comparative study based on the Monaco TPS has been conducted between non-coplanar VMAT and non-coplanar fixed-field IMRT for HA-WBRT+SIB. The purpose of this work is to explore their advantages and provide feasible treatment plans for patient treatment.

## 2 Materials and methods

### 2.1 Patient data

In total, 22 patients with brain metastases who received radiotherapy at our hospital from June 2022 to October 2023 were retrospectively enrolled, including 10 men and 12 women, aged 27 to 83 years. None of the patients had metastases invading the hippocampus. The basic characteristics of the 22 patients are shown in **Table 1**.

**Table 1:** Basic patient characteristics (n= 22).

Number	Sex	Age	Primary tumor	Number of metastatic lesions	Metastatic lesions total volume(cm^3^)	Hippocampal volume(cm^3^)	Hippocampal avoidance (HA) volume(cm^3^)	PTV-brain-SIB volume(cm^3^)	HA volume as a percentage of total brain volume(%)
1	Female	38	Breast	2	50.8	8.9	38.3	1275.2	2.81%
2	Female	75	Lung	5	28.8	6.7	32.0	1071.7	2.83%
3	Male	54	Lung	1	8.8	8.3	36.3	1443.7	2.44%
4	Female	54	Lung	3	28.4	7.5	34.9	1004.6	3.27%
5	Female	60	Lung	1	4.2	6.3	32.0	1137.3	2.73%
6	Male	58	Lung	2	45.0	7.4	32.9	1398.2	2.23%
7	Male	72	Lung	11	40.4	8.2	37.0	1245.3	2.80%
8	Male	55	Lung	6	108.1	7.5	34.6	1180.8	2.62%
9	Female	66	Sigmoid	2	20.3	7.0	33.3	1234.9	2.59%
10	Female	63	Rectum	1	21.6	4.4	31.0	1103.7	2.69%
11	Female	52	Lung	3	16.7	3.3	21.3	1134.7	1.82%
12	Male	40	Lung	7	60.7	6.6	32.9	1265.8	2.42%
13	Male	65	Lung	4	21.5	10.8	44.2	1305.5	3.22%
14	Female	27	Lung	2	20.3	5.7	29.2	1335.5	2.11%
15	Female	83	Lung	2	88.6	5.2	27.2	1065.8	2.31%
16	Male	69	Lung	1	14.8	7.8	34.8	1437.9	2.34%
17	Male	75	Lung	3	90.8	6.0	29.1	1348.2	1.99%
18	Female	55	Lung	13	34.0	6.6	34.8	1141.8	2.88%
19	Male	36	Brain	1	30.7	4.1	25.9	1354.6	1.84%
20	Male	77	Ascending colon	1	20.1	8.1	36.7	1312.1	2.68%
21	Female	46	Lung	2	39.2	9.5	38.9	1231.2	2.97%
22	Female	48	Breast	1	197.1	7.3	35.0	1141.0	2.55%

The study was approved by the ethics committee of Gaozhou People’s Hospital (GYLLYJ-2022111). Since it is a retrospective study that presents no risk to the participants’ health or economic well-being, the ethics committee of Gaozhou People’s Hospital granted an exemption from obtaining informed consent.

### 2.2 Computed tomography simulation and target delineation

The patients were immobilized in a supine position using a head and neck thermoplastic mask and immobilization bag. All the patients underwent computed tomography (CT) imaging acquisition using a large-bore CT simulator (Siemens AG, Forchheim, Germany), with a scan slice thickness and slice interval of 1.5 mm. The scanning range extended from the cranial apex to the third cervical vertebra. Additionally, each patient underwent contrast-enhanced T1-weighted magnetic resonance imaging (MRI) (Siemens AG, Forchheim, Germany) with a slice thickness of 1.5 mm. CT and MR images were imported to the Monaco TPS (Elekta, Crawley, England) for fusion. Following the RTOG 0933 delineation guideline, the hippocampus was delineated, expanding the total hippocampus by 5 mm in all directions to create a HAR. Other OARs were also delineated, including whole-brain tissue, brainstem, lens, eyes, optic nerves, optic chiasm, pituitary, and inner ears. An enhanced lesion was defined by the gross tumor volume (GTV), which was expanded by 3 mm in all directions to form the planning gross tumor volume (PGTV). The whole-brain tissue excluding the HAR, and subtracting the PGTV was defined as the whole-brain planning target (PTV-brain-SIB).

### 2.3 Treatment planning

The prescription dose for all patients was 30 Gy to the PTV-brain-SIB in 15 fractions (2 Gy per fraction), and 50 Gy to the PGTV in 15 fractions (3.33 Gy per fraction). Both targets were treated simultaneously. The PGTV requires 100% of the prescription dose to cover at least 95% of the volume (V_50_≥95%).

Two plans were created for each patient, namely, non-coplanar VMAT and IMRT. Both plans were optimized using the Monte Carlo dose calculation algorithm base Monaco 5.4 TPS on an Axesse linear accelerator (Elekta, Crawley, Sweden) with an Agility multileaf collimator, using a 6 MV photon beam. Non-coplanar VMAT included two fields, with the first field being a 360°coplanar rotation arc starting from 180°, with an increment of 20°, the collimator angle was set at 0°, and the treatment couch angle was set at 0°. The second non-coplanar field couch was set at 270°, while the gantry started at 330°, with a rotational span of 210° and an increment of 15°. The collimator angle was set at 0°.

Non-coplanar fixed-field IMRT used nine fields with gantry angles at 5°, 55°, 135°, 165°, 215°, 270°, 315°, 70°, and 30°, where the treatment couch angle for the 70° and 30° fields was 270°, and for the other seven fields couch angle was 0°. The collimator angle for all nine fields was 315°. The choice of field angle and couch angle was based on experience in daily practice.

**Figure 1:**
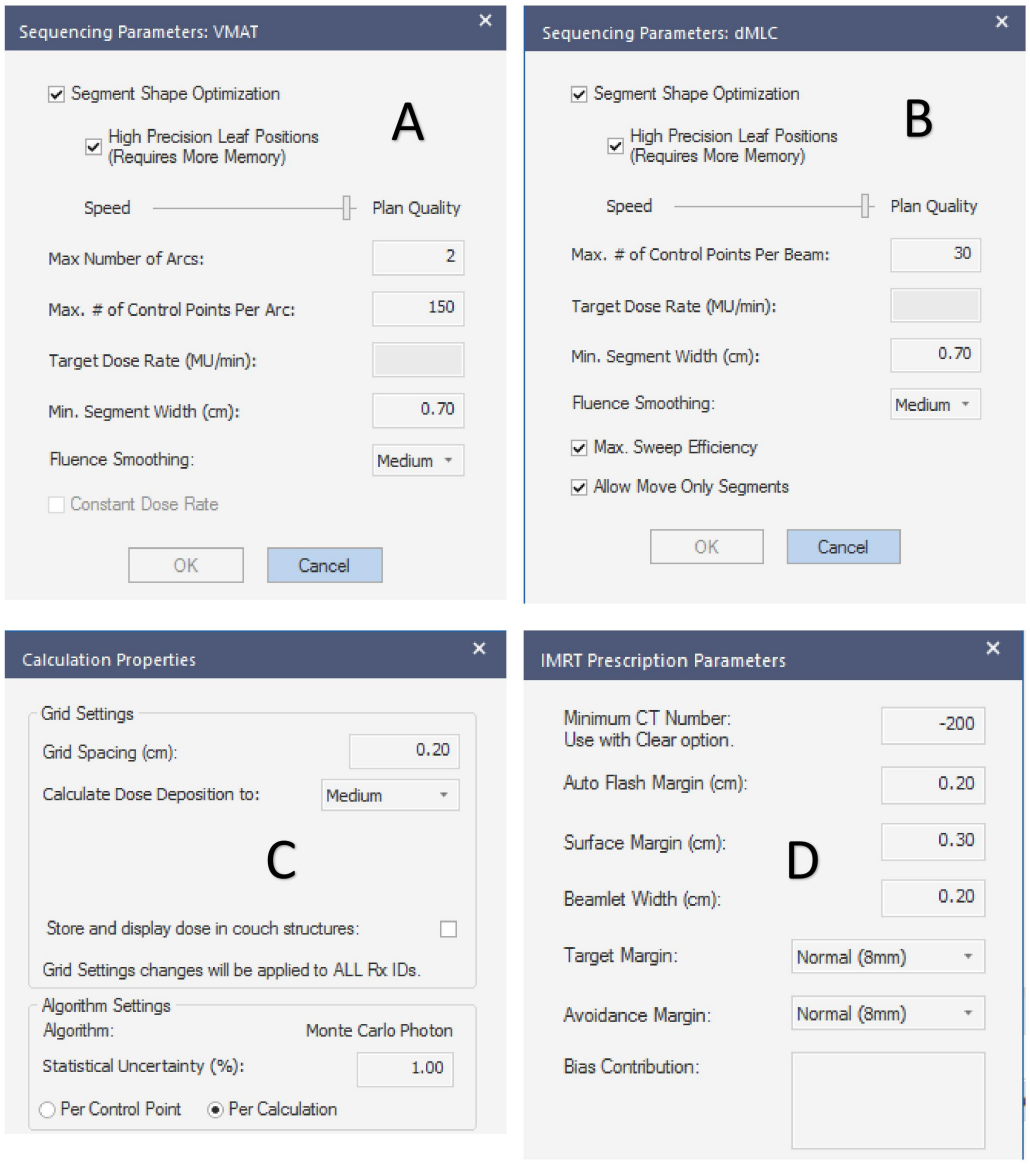
Plan setting parameters. **(A–D)** are the sequencing parameters of the VMAT plan, sequencing parameters of the IMRT plan, the calculation properties of the planned dose deposition, and the prescription dose parameters, respectively.

The planned sequencing parameters, dose deposition calculation properties, and prescription parameters are shown in **Figure 1**.

The dose limits for the targets and OARs for both plans are shown in **Table 2**. The same optimization functions and parameters were used for both plans and optimization templates were created to save planning time, as shown in **Table A1**.

**Table 2:** Dose criteria for the targets and organs at risk.

Structure	Dose limits
PGTV	V_55_<5%, V_50_≥95%
PTV-brain-SIB	V_30_≥90%, D_98%_≥25 Gy
Left and right hippocampus	D_100%_<9 Gy(D_100%_>10 Gy not acceptable), D_max_<16 Gy(D_max_>17 Gy not acceptable)
Left and right optic nerves	D_max_<37.5 Gy
Optic chiasm	D_max_<37.5 Gy
Left and right lens	D_max_<8 Gy
Left and right eyes	D_max_<37.5 Gy
Brainstem	D_max_<37.5 Gy
Left and right inner ears	D_max_<37.5 Gy
Pituitary	D_max_<37.5 Gy

V_x_ is the volume of the region of interest (ROI) when the dose received is x Gy, D_x%_ is the dose corresponding to x% of the ROI volume, and D_max_ is the maximum dose.

### 2.4 Dosimetric comparison of target and OARS

A comparison was conducted between the VMAT and IMRT plans for the patient cohort. For the PGTV, V_50_ and V_55_, the heterogeneity index (HI), and the conformity index (CI) were compared. For the PTV-brain-SIB, D_98%_, V_30_, the HI, and the CI were compared. The HI was calculated as D_5%_/D_95%_ (8). A smaller HI indicates a more uniform dose distribution in the target. The CI was calculated as V^2RX^/(TV*V_RI_) (23), where V_RX_ is the volume covered by the prescription dose in the target, TV is the volume of the target, and V_RI_ is the volume covered by the prescription dose. The CI ranges from 0 to 1. A higher CI indicates a more conformal dose distribution in the target. For the hippocampus, D_100%_ and the maximum dose (D_max_) were compared, while for other OARs, the D_max_ was compared (brainstem, lens, eyes, optic nerves, optic chiasm, inner ears, and pituitary).

### 2.5 Deliverability of the two plans

To examine the deliverability, the monitor units (MUs) and beam-on time for the two plans were compared. All plans were delivered in quality assurance (QA) mode, and the beam-on time was recorded using a calibrated stopwatch. The beam-on time only considered the beam irradiation time without considering the gantry rotation time between arcs or fields.

### 2.6 Pre-treatment quality assurance

Pre-treatment plan verification was performed using ArcCheck (SUN NUCLEAR, California, US). We composited all the fields and did not reset the gantry to 0° but reset the couch to 0°. Gamma analysis was conducted on the dose distribution of the planned and measured data, with evaluation criteria of 3%/3 mm and a 10% dose threshold (TH). A pass rate of at least 95% was considered passed. A more stringent gamma analysis was performed using 3%/2 mm or 2%/2 mm to test the two techniques.

### 2.7 Statistical analysis

Statistical analysis was conducted using SPSS 17.0 (IBM, USA). Normality was tested on the data, and for parameters conforming to a normal distribution, data were expressed as mean ± standard deviation ( $\bar{x}\pm s$ ). Those that did not fit the normal distribution were tested using the non-parametric Friedman test for multi-correlated samples, and quantitative data were expressed as medians and 25% and 75% percentiles (P25 and P75). A paired t-test was used for comparisons and a *p*-value<0.05 was considered statistically significant.

## 3 Results

### 3.1 Target dose comparison

**Figure 2:**
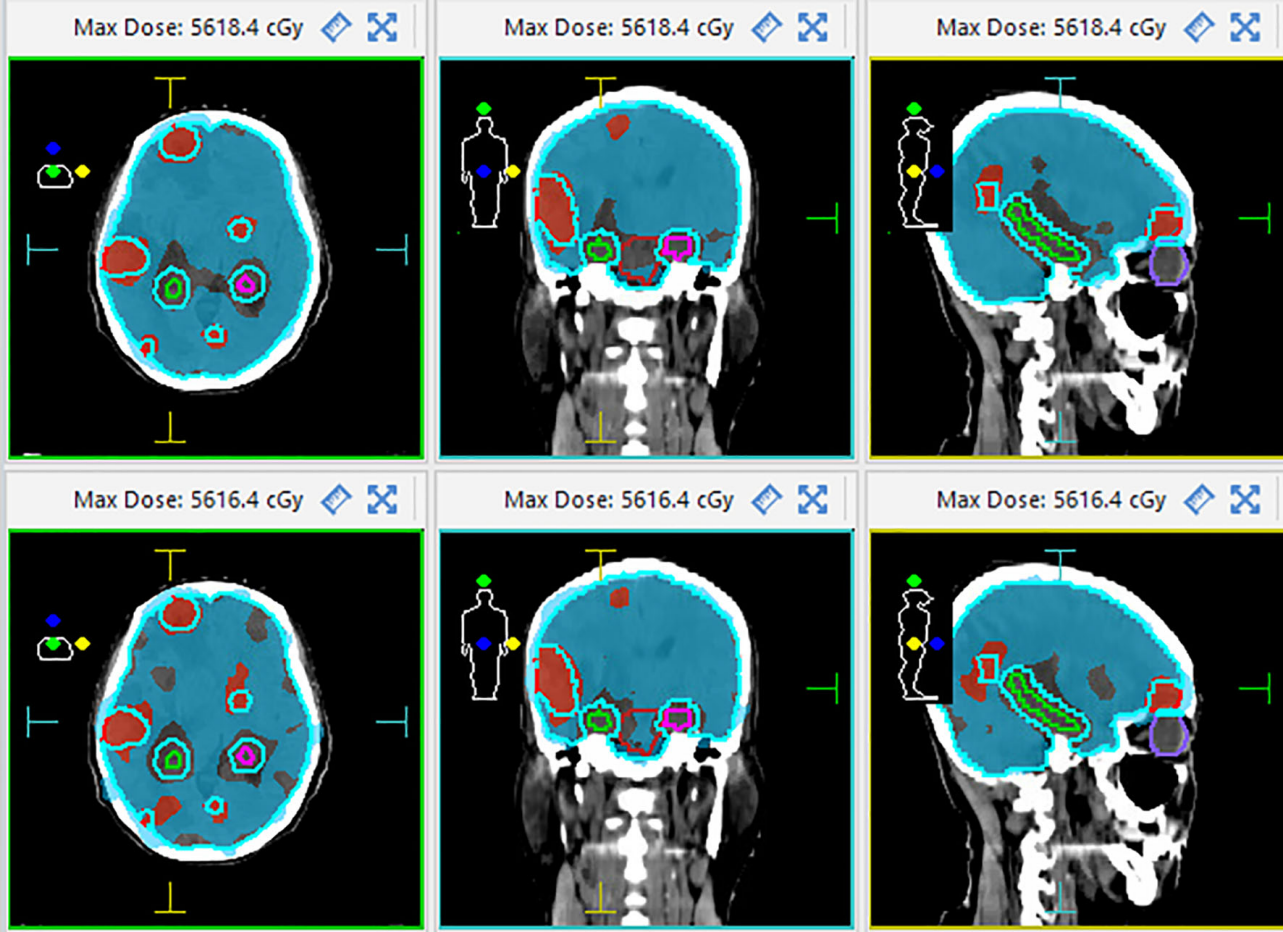
The dose distribution of the VMAT (upper) and IMRT (down) plans in cross-section (left), coronal (middle), and sagittal plane (right) for a typical patient. The red area represents the coverage with an isodose of 50 Gy and the blue area represents the coverage with an isodose level at 30 Gy.

**Figure 3:**
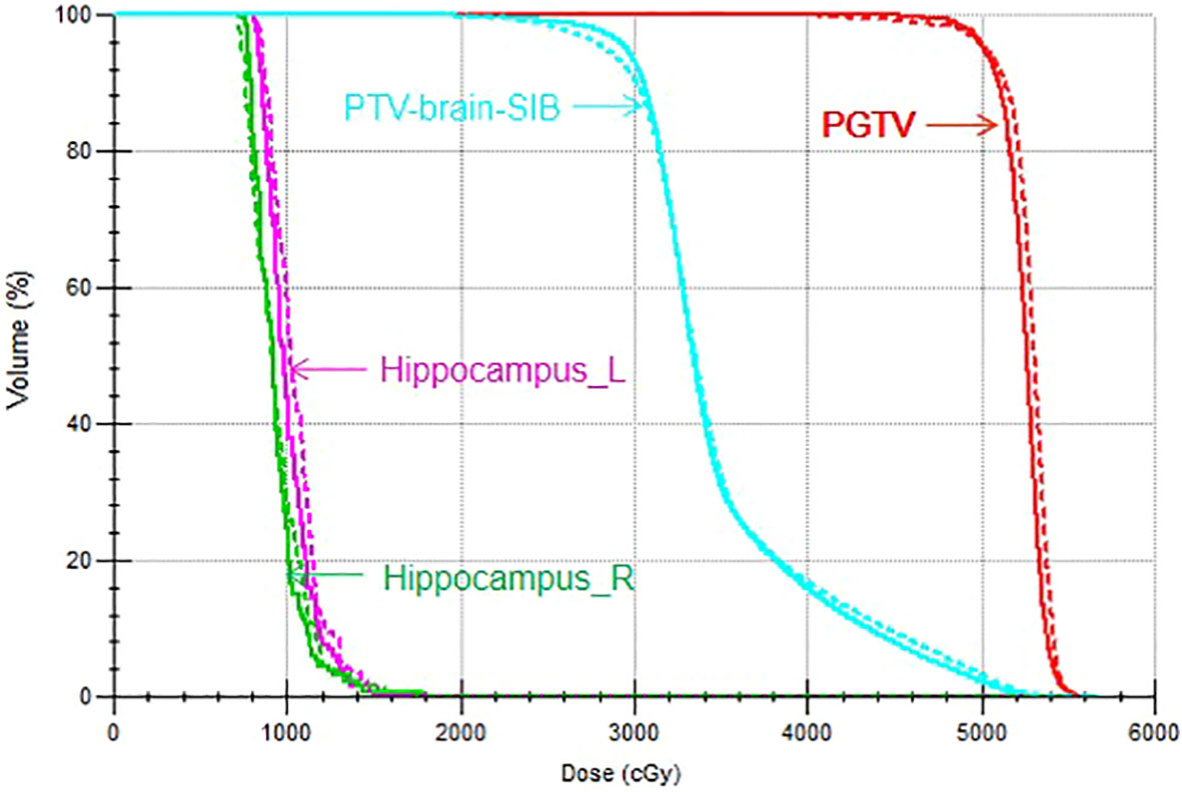
DVH of the VMAT and IMRT plans for a representative patient. The solid line indicates the VMAT plan and the dotted line indicates the IMRT plan.

The target dose of both plans for the 22 patients met the clinical goals, as shown in **Table 2**. For the PGTV, there was no significant difference in V_50_, V_55_, D_max_, the HI, and the CI between VMAT and IMRT (*p* > 0.05). For the PTV-brain-SIB, there was no significant difference in D_98%_ between IMRT and VMAT (*p* = 0.103); VMAT significantly increased the V_30_ for PTV-brain-SIB (*p*< 0.001), reduced the HI (*p* = 0.003), and increased the CI (*p*< 0.001), as shown in **Table 3**. The dose distribution for a typical patient is illustrated in **Figure 2**, where the VMAT plan showed a better coverage of the 30 Gy dose, and the IMRT plan had larger cold spots. The dose-volume histogram (DVH) for a representative patient is shown in **Figure 3**.

**Table 3:** PGTV and PTV-brain-SIB dose comparison between the IMRT and VMAT plans for the patient cohort.

Structure	Parameter	IMRT	VMAT	Difference (%)	*p-*value
PGTV	V_50_ (%)	97.26 ± 1.43	97.46 ± 1.62	-0.01 ± 0.01	0.350
	V_55_ (%)	1.35 ± 1.19	1.35 ± 0.97	0.26 ± 1.33	0.983
	D_max_ (cGy)	5582.8 ± 62.8	5578.5 ± 46.3	0.08 ± 1.11	0.749
	HI	1.07 ± 0.01	1.07 ± 0.01	0.13 ± 0.82	0.480
	CI	0.68 ± 0.10	0.69 ± 0.09	-1.37 ± 6.21	0.341
PTV-brain-SIB	D_98%_ (cGy)	2696.2 ± 82.9	2724.5 ± 71.2	-1.01 ± 2.90	0.103
	V_30_ (%)	92.09 ± 1.25	93.32 ± 1.23	-0.01 ± 0.01	*<0.001*
	HI	1.46 ± 0.17	1.44 ± 0.16	1.44 ± 2.03	*0.003*
	CI	0.77 ± 0.05	0.81 ± 0.04	-4.16 ± 2.44	*<0.001*

p value denotes the results of paired t-test between IMRT and VMAT plans. The italicized values indicated p value is less than 0.05.

### 3.2 Organs-at-risk dose comparison

The dose comparison of OARs for both plans is presented in **Table 4**. There were no significant differences in the D_max_ for the brainstem, left and right lenses, optic chiasm, pituitary, and left and right hippocampus between the two plans (*p*>0.05). VMAT, relative to IMRT, significantly reduced the D_max_ for the left eye and right eye (*p*< 0.001), significantly increased the D_max_ for the right inner ear (*p* = 0.010), and had no significant difference in the D_max_ for the left inner ear (*p* = 0.458). VMAT significantly reduced the D_max_ for the left optic nerve (*p*=0.006) but significantly increased the D_max_ for the right optic nerve (*p* = 0.001) compared to IMRT. There were no significant differences in the D_max_ for the left and right hippocampus between VMAT and IMRT (*p* > 0.05), but VMAT significantly increased the D_100%_ for both the left and right hippocampus (*p*< 0.05).

**Table 4:** Comparison of the OAR doses between the IMRT and VMAT plans.

Structure	Parameter	IMRT	VMAT	Difference (%)	*p*-value
Brainstem	D_max_ (cGy)	3564.7 ± 511.7	3551.6 ± 513.2	0.40 ± 2.60	0.493
Left eye	D_max_ (cGy)	2625.1 ± 363.1	2320.7 ± 271.3	13.26 ± 11.23	*<0.001*
Right eye	D_max_ (cGy)	2672.8 ± 374.0	2380.5 ± 442.0	13.54 ± 12.52	*<0.001*
Left lens	D_max_ (cGy)	620.8 ± 32.6	630.2 ± 28.5	-1.39 ± 5.15	0.194
Right lens	D_max_ (cGy)	630.5 ± 52.3	646.7 ± 32.8	-2.34 ± 8.26	0.185
Left inner ear	D_max_ (cGy)	3190.2 ± 105.7	3160.8 ± 123.1	1.10 ± 5.90	0.458
Right inner ear	D_max_ (cGy)	3073.0 ± 170.2	3152.1 ± 169.9	-2.43 ± 4.08	*0.010*
Left optic nerve	D_max_ (cGy)	3070.2 ± 125.8	2978.3 ± 130.0	3.20 ± 4.72	*0.006*
Right optic nerve	D_max_ (cGy)	2724.5 ± 296.2	2943.5 ± 208.4	-7.30 ± 9.54	*0.001*
Optic chiasm	D_max_ (cGy)	3246.8 ± 108.8	3215.2 ± 110.0	1.03 ± 3.30	0.183
Pituitary	D_max_ (cGy)	3034.7 ± 115.4	3053.9 ± 67.0	-6.03 ± 3.74	0.445
Left hippocampus	D_100%_ (cGy)	757.2 ± 49.6	778.8 ± 59.1	-2.59 ± 5.00	0.018
	D_max_ (cGy)	1554.1 ± 249.4	1515.3 ± 130.1	2.14 ± 7.00	0.186
Right hippocampus	D_100%_ (cGy)	742.7 ± 62.6	790.0 ± 59.1	-5.96 ± 4.07	*<0.001*
	D_max_ (cGy)	1504.3 ± 129.7	1496.6 ± 96.7	0.49 ± 5.17	0.647

p value denotes the results of paired t-test between IMRT and VMAT plans. The italicized values inidicate pvalue is less than 0.05.

### 3.3 Deliverability of the plans

The average MUs for non-coplanar IMRT and non-coplanar VMAT were 1174 MU (990~1592) and 1326 MU (1112~1660), respectively. IMRT significantly reduced the planned MU compared to VMAT (*p*<0.001). VMAT significantly reduced the beam on time with a mean reduction of 41 seconds (294.1 ± 16.4 seconds vs. 335.8 ± 34.9 seconds, *p*<0.001) compared to IMRT.

### 3.4 Pre-treatment plan quality assurance

The mean gamma passing rates for the VMAT and IMRT plans were 99.4% and 99.5%, respectively, with the 3%/3 mm and 10% threshold criteria, and there was no significant difference (*p*=0.125). However, with the 3%/2 mm, and 2%/2 mm criteria, the passing rate for IMRT was higher than VMAT (99.1 ± 0.56 vs 98.5 ± 0.86, 97.6 ± 1.05 vs 96.0 ± 1.41) with a mean increase of 0.6% (*p*=0.013) and 1.7% (*p*<0.001), respectively.

## 4 Discussion

Several publications have studied HA-WBRT or HA-WBRT+SIB based on different techniques or different linear accelerators (10, 15–17, 21, 24–27). However, there have been no dose comparison studies on HA-WBRT+SIB using non-coplanar VMAT and non-coplanar IMRT based on the Monaco TPS. Different from previous studies (10, 15, 16), the current research protected the brainstem, inner ear, pituitary, and hippocampus while reaching the dose coverage of the target. The D_max_ of the left hippocampus of the current study was 1554.1 ± 249.4 Gy for IMRT and 1515.3 ± 130.1 Gy for VMAT, respectively. The D_max_ of the right hippocampus was 1504.3 ± 129.7 Gy for IMRT and 1496.6 ± 96.7 Gy for VMAT, respectively, which were much lower than that of the study of Johannes Kraft (20) and XIE XIN (22), and the HAR in this study was only a 5 mm outward expansion around the hippocampus. Yu Xiao et al. used the Pinnacle TPS to improve the existing non-coplanar VMAT HA-WBRT plan, and compared the dosimetric differences between the improved non-coplanar VMAT plan and the traditional non-coplanar and coplanar VMAT plans in HA-WBRT (28). For the D_max_ and D_100%_ in hippocampal tissue, the improved non-coplanar VMAT could be controlled at approximately 14.37 Gy and 8.40 Gy, respectively, which were significantly smaller than the traditional non-coplanar and coplanar plans (*p*<0.05). However, the improved non-coplanar VMAT plan was too complicated, with a total of six arcs and four couch angles (270°, 315°, 45°, and 0°), so the treatment efficiency was low. Shao Wei et al. investigated the dosimetric differences between VMAT with a flattening filter (FF) and flattening filter free (FFF) in HA-WBRT using four half arcs with a prescribed dose of 30 Gy in 10 fractions. The D_max_ of the hippocampus in the FF-VAMT and FFF-VMAT plans was 16.46 ± 0.56 Gy and 15.13 ± 0.38 Gy, respectively, and the D_100%_ was 7.72 ± 0.28 Gy and 7.12± 0.34 Gy, respectively. The results of the current study are comparable to the two plans, but their study did not have SIB (29).

This study was conducted based on the Monaco TPS and the Elekta Axesse linear accelerator, and a template for field setup and optimization functions was created to save planning time. The most important factor in successful HA-WBRT+SIB planning is the TPS and field setup and the optimization of function criteria. While some plans required fine-tuning for specific cases, such as an SIB region that is too close to the hippocampus (<3 mm) or a large number and volume of brain metastases, the optimization function template provided in this study met the planning requirements for the majority of patients. Among the 22 patients, only one patient had a D_max_ to the left hippocampus that exceeded 17 Gy (26.21 Gy for IMRT and 20.52 Gy for VMAT, respectively). This was due to its proximity to the SIB target, which was only 1 mm from the lesion at 50 Gy. This differs from previous studies that only provided field settings without essential optimization functions (8, 30–32). This study provides detailed planning optimization parameter settings for the clinical implementation of this treatment technique. It protects the pituitary gland, inner ear, and hippocampus. The two plans in this study showed no significant differences in the dose distribution for the target PGTV with SIB. However, VMAT significantly increased the V_30_, conformity, and uniformity for PTV-brain-SIB (*p*<0.05). The plans exhibited no significant differences in the D_max_ to the hippocampus (*p*>0.05), but IMRT significantly reduced the D_100%_ for the hippocampus, with a lower MU compared to VMAT. Although IMRT reduced the MU, it did not reduce the beam on time, resulting in a time advantage. The reason may be that VMAT can beam on continuously during rotation. When considering the idling time of the gantry, IMRT requires more treatment time than VMAT, so from a cost/benefit perspective, VMAT can treat more patients than IMRT in the same amount of time. IMRT had a significantly higher gamma passing rate than VMAT under the 3%/2 mm and 2%/2 mm criteria, possibly due to the reduced MU that reduced the linear accelerator’s head leakage.

Collision avoidance is an important issue in a non-coplanar plan, and the Monaco TPS provides the Room’s Eye View function to view the gantry, couch, lighting, decorations, axes, and the active treatment beam in a room to make sure that the patient is in a safe position. Acquiring the patient’s CBCT will be a problem when the non-coplanar plan is implemented for treatment; we can only acquire CBCT when the couch angle is at 0°, so it is important to make sure that the center of couch rotation accuracy meets the requirements so that the dose distribution is as expected.

The current study has several limitations. First, it was a retrospective study comparing dosimetry, did not compare biology such as probability of tumor control and probability of normal tissue complications, and was not a prospective study with clinical outcomes. Therefore, the advantages and disadvantages of the two techniques need to be determined by long-term patient follow-up and based on extensive case practice. Second, there were no comparisons of other IMRT techniques such as helical tomography and proton and carbon ion therapy. Third, the correlation between dose limitation in the hippocampus and symptom reduction in patients has not been adequately demonstrated. Fourth, the TPS and dose calculation algorithms used for planning can also have an impact on outcomes. Thus, different TPSs such as Varian’s Eclipse and RaySearch’s RayStation and different algorithms such as anisotropy analysis algorithm and pencil beam need to be further investigated. Fifth, the measurement data for all QA measurements in this study were not completed during the same time period, and the deviation of the dose output from the machine each time may have an effect on the QA results. Sixth, the control point limits of the two plans were not uniform, and different control points may have significant effects on dose distribution.

## 5 Conclusion

This study performed a dosimetric comparison of non-coplanar VMAT and non-coplanar IMRT for HA-WBRT+SIB in brain metastasis patients on the Monaco TPS. Both plans demonstrated clinically acceptable results for hippocampal protection. VMAT had advantages in target dose distribution and treatment efficiency, while IMRT protected the hippocampus better and reduced the machine monitor units.

## Data Availability

The raw data supporting the conclusions of this article will be made available by the authors, without undue reservation.
