# Supplementary material for: A dosimetric comparison of non-coplanar volumetric modulated arc therapy and non-coplanar fixed field intensity modulated radiation therapy in hippocampus-avoidance whole-brain radiation therapy with a simultaneous integrated boost for brain metastases
Source: Front Oncol. 2025 Jan 23;14:1428329. doi: 10.3389/fonc.2024.1428329 (PMC11799273; doi:10.3389/fonc.2024.1428329)
Supplement: Supplementary file 1 [file Table1.docx]

Supplementary files：

Appendix

Table A1 Parameters of the optimization functions used for plan optimization

| Structure | Cost function | Parameters setting |
| --- | --- | --- |
| PGTV | Target Penalty | Prescription(cGy)=5000，minimum volume=98% |
|  | Quadratic Overdose | Maximum Dose(cGy)=5200，RMS Dose Excess=150 cGy |
|  | Underdose DVH | Objective Dose(cGy)=5000，Minimum Volume(%)=95 |
| Hippocampus_L | Quadratic Overdose | Maximum Dose(cGy)=1300，RMS Dose Excess(cGy)=30 |
|  | Parallel | Reference Dose(cGy)=720，Mean Organ Damage(%)=60，  Power Law Exponent=4 |
| Hippocampus_R | Quadratic Overdose | Maximum Dose(cGy)=1300，RMS Dose Excess(cGy)=30 |
|  | Parallel | Reference Dose(cGy)=720，Mean Organ Damage(%)=60，  Power Law Exponent=4 |
| PTV-brain-SIB | Target Penalty | Prescription(cGy)=3100，Minimum Volume(%)=90，  Shrink Structures(PGTVm，include=yes，Margin=0.00 cm) |
|  | Quadratic Overdose | Maximum Dose(cGy)=5000，RMS Dose Excess=20 cGy  Shrink Structures(PGTVm，include=yes，Margin=0.10 cm) |
|  | Quadratic Overdose | Maximum Dose(cGy)=3600，RMS Dose Excess=2 cGy  Shrink Structures(PGTVm，include=yes ，Margin=1.60 cm) |
|  | Underdose DVH | Objective Dose(cGy)=3000，Minimum Volume(%)=90，  Shrink Structures(PGTVm，include=yes，Margin=0.00 cm) |
| Lens_L | Serial | Equivalent Uniform Dose(cGy)=600，Power Law Exponent=20 |
| Lens_R | Serial | Equivalent Uniform Dose(cGy)=600，Power Law Exponent=20 |
| OpticChiasm | Serial | Equivalent Uniform Dose(cGy)=3000，Power Law Exponent=20 |
| OpticNerve_L | Serial | Equivalent Uniform Dose(cGy)=3000，Power Law Exponent=20 |
| OpticNerve_R | Serial | Equivalent Uniform Dose(cGy)=3000，Power Law Exponent=20 |
| Eye_L | Serial | Equivalent Uniform Dose(cGy)=3000，Power Law Exponent=20 |
| Eye_R | Serial | Equivalent Uniform Dose(cGy)=3000，Power Law Exponent=20 |
| BrainStem | Serial | Equivalent Uniform Dose(cGy)=3000，Power Law Exponent=20 |
| Pituitary | Serial | Equivalent Uniform Dose(cGy)=3000，Power Law Exponent=20 |
| InnerEar_L | Serial | Equivalent Uniform Dose(cGy)=3000，Power Law Exponent=20 |
| InnerEar_R | Serial | Equivalent Uniform Dose(cGy)=3000，Power Law Exponent=20 |
| patient | Quadratic Overdose | Maximum Dose(cGy)=3050，RMS Dose Excess=50 cGy  Shrink Structures (PGTVm，include=yes，Margin=0.00 cm；ptv-brain-SIB，include=yes，Margin =0.10) |
|  | Quadratic Overdose | Maximum Dose (cGy)=5400，RMS Dose Excess=2 cGy |
